# Semaglutide treatment of hypothalamic obesity – a real-life data study

**DOI:** 10.1007/s11102-024-01429-5

**Published:** 2024-08-09

**Authors:** Mathilde Svendstrup, Aase Krogh Rasmussen, Caroline Kistorp, Marianne Klose, Mikkel Andreassen

**Affiliations:** Department of Endocrinology, Rigshospitalet, Copenhagen University Hospital, Copenhagen, Denmark

**Keywords:** Hypothalamic obesity, Hypothalamic tumors, Craniopharyngiomas, GLP-1 analogue treatment, Weight loss

## Abstract

**Purpose:**

Patients with tumors involving the hypothalamic region are at high risk of developing morbid obesity due to disturbances in the appetite regulative nuclei in hypothalamus. We evaluated the effect of the Glucagon-like peptide 1 (GLP-1) analogue semaglutide in patients with hypothalamic obesity.

**Methods:**

We recorded weight changes from real-time data before and after treatment with semaglutide in patients with hypothalamic obesity from our outpatient clinic at the Department of Endocrinology at Rigshospitalet, from September 2020 to November 2023.

**Results:**

A total of 26 patients were included in this study (15 females, median age at initiation of semaglutide was 52 (range 18–65) years). Body mass index (BMI) at initial diagnosis was median 25 (range 20–38) kg/m^2^ while BMI at initiation of semaglutide was median 38 (range 28–58) kg/m^2^. All but one patient lost weight during semaglutide treatment with a mean weight loss of 13.4 kg (95% CI 10.3–16.5 kg, *p* = < 0.001) after 12 months corresponding to a loss in BMI of 4.4 kg/m^2^ (95% CI 3.4–5.4 kg/m^2^, *p* = < 0.001) with a median dosage of semaglutide of 1.6 (range 0.5–2.5) mg. Fifteen patients (58%) lost more than 10% and two patients (8%) lost more than 20% of initial body weight, respectively.

**Conclusion:**

Treatment with semaglutide shows promising results in reducing body weight in patients with acquired hypothalamic obesity. Whether the weight reduction remains stable after long time follow-up needs further investigation.

## Introduction

The hypothalamus nests key nuclei crucial for regulation of basal metabolic functions like sleep, temperature regulation, appetite and energy homeostasis [1, 2]. Mono-genetic diseases affecting the hypothalamic appetite center may cause early onset of massive obesity [3]. A similar obesity pattern is seen after traumatic lesions of the hypothalamic area [4]. Hypothalamic obesity (HO) is characterized by a disrupted appetite regulation and extreme food-seeking behavior in combination with reduced energy expenditure [5, 6]. Further, patients with hypothalamic lesions might experience sleep disorders, cognitive deficits as impaired short time memory, and pituitary insufficiency further indirectly affecting body weight and complicating weight loss.

Craniopharyngiomas are histologically benign tumors in the sellar/parasellar region originating from cystic embryonic malformations. Craniopharyngiomas often extent to the medial hypothalamic nuclei, impacting important hormone regulatory sites such as the arcuate, ventromedial and dorsomedial nuclei, thereby affecting hormone production in the pituitary gland [7]. Other tumors potentially affecting the hypothalamic area are germinomas, gliomas and large pituitary adenomas.

Patients suffering from craniopharyngiomas and other hypothalamic tumors often experience a massive weight gain before or within short time (months) of diagnosis, a result of either the tumor itself or subsequent surgical and/or irradiation therapy in the area.

GLP-1 analogues promote weight loss by increasing satiety and reducing gastric emptying in obese patients with or without Type 2 diabetes [8, 9]. Some studies have also indicated an increase in energy expenditure during treatment with GLP-1 analogues [10, 11]. GLP-1 analogues exert their central effects at receptors in the hypothalamic nuclei as well as at extra-hypothalamic receptors in the hindbrain and the vagal nerve [12]. Successful treatment of HO using GLP-1 analogues has been suggested in case reports and in a few intervention-studies employing different type and dosages of GLP-1 analogues. Most studies used daily injection with liraglutide or exendin-4 [13–16]. Two other studies – a case report and one randomized-controlled trial (RCT) – has investigated the effect of weekly injection with long-acting exenatide or dulaglutide [17–19]. To our knowledge, only one published case report exists on semaglutide treatment for hypothalamic obesity in humans [20].

In populations with overweight with and without diabetes, once-weekly injection with semaglutide has shown to be superior to liraglutide regarding weight loss [21–23].

Since 2020, the Department of Endocrinology at Rigshospitalet, Denmark, has treated HO patients with semaglutide. In this paper, we report the weight change outcome from this treatment.

## Materials and methods

We recruited patients who started treatment for hypothalamic obesity with semaglutide between September 2020 and April 2023. Out of 26 patients, nine had previously been treated with liraglutide before the beginning of the observation period. Hypothalamic obesity was considered in patients diagnosed with suprasellar tumors and concurrent presence of obesity (body mass index (BMI) ≥ 30 kg/m^2^). In two out of 26 cases, semaglutide treatment was started before the presence of obesity (at BMI 28 and 29 kg/m^2^, respectively). In these two subjects, semaglutide treatment was started after discussion in the medical team based on an abrupt weight gain in the patient related to the hypothalamic injury as judged by the treating physician. We followed patients until 24 months of semaglutide treatment, stop of treatment (different causes), death (1 patient) or November 2023. The dosage of semaglutide was titrated to a target dose of 2.5 mg/day or lower on an individual estimation by the treating clinician. Patients were instructed in the injection technique by a nurse, but no additional dietician counselling or other weight loss support was added to the treatment with semaglutide. Data of body weight were collected from patient files as a mixture of measured and self-reported data. The patients were concurrently substituted with relevant hormones according to their individual disturbances in pituitary function. They were treated according to standard care and were generally well-substituted with the relevant hormones. Further details are given in the results section.

### Statistical analysis

All analyses were performed using R version 4.0.2. Baseline data are presented as number (percent) or median (range) due to non-normally distributed data and the small size of the data set.

The results of weight change are expressed in kg, in percentage and in change in BMI from baseline to 3, 6, 12, 18 and 24 months of treatment, respectively.

To statistically evaluate the effect of semaglutide on change in body weight (in kg and %) and BMI, we employed a general linear mixed model for repeated measurements with random intercept and random slope and with time as a factor (3, 6, 12, 18 and 24 months, respectively). Differences in response between pre-specified groups (men vs. women, diabetes vs. no diabetes, previous liraglutide vs. no previous GLP-1 analogue treatment, and time from diagnosis to treatment above or below two years, respectively) was tested by adding an interaction term between time and the specified group to the model. Assumptions of the model was checked by visual inspection.

## Results

A total of 26 patients started treatment with semaglutide (15 females, median age at initiation of semaglutide was 52 (range 18–65) years). Median body weight at initial diagnosis of the hypothalamic tumor was 80 (range 50–140) kg (median BMI 25 (range 20–38) kg/m^2^) which increased over a median period of 4 (range 0–31) years to 116 (range 76–175) kg (median BMI 38 (range 28–58) kg/m^2^) before initiation of semaglutide (Table 1). Nine patients had previously been treated with liraglutide but was converted to semaglutide treatment once it became available in Denmark. The remaining 17 patients commenced treatment directly with semaglutide. After the up-titration phase, the median dose of semaglutide at the end of the observation period was median 1.6 (range 0.5–2.5) mg weekly.

**Table 1:** Baseline characteristics

**Number**	26
- Female	15 (58%)
- Male	11 (42%)
**Age at initial diagnosis of hypothalamic tumor (years)**	43 (1;60)
**Diagnosis**:	
- Craniopharyngioma	15 (58%)
- Pituitary adenoma (non-producing)	3 (12%)
- Acromegaly	2 (8%)
- Germinoma	1 (4%)
- Spindle cell oncocytoma	1 (4%)
- Neuroendocrine cerebral metastasis	1 (4%)
- Pilocystic astrocytoma	1 (4%)
- Supratentorial primitive neuroectodermal tumor	1 (4%)
- Opticus glioma	1 (4%)
**Tumor size (mm)**	30 (15;50)
**Operation (Yes)**	25 (96%)
**Re-operation (Yes)**	6 (23%)
**Irradiation (Yes)**	7 (27%)
**Remaining tumor (Yes)**	10 (38%)
**Number of affected hormone axes (N)**	4 (0;5)
**Substitution treatment (Yes)**	25 (96%)
- Hydrocortisone	18 (69%)
- Growth hormone	18 (69%)
- Thyroid hormone	25 (96%)
- Sex hormone	18 (69%)
- Anti-diuretic hormone	17 (65%)
**Co-existing diabetes mellitus (Yes)** ^**1**^	7 (27%)
**Previous liraglutide treatment (Yes)**	9 (35%)
**Age at start of semaglutide**	52 (18;65)
**Time from diagnosis to start of semaglutide (years)**	4 (0;31)
**Body weight at diagnosis (kg)**	81 (50;140)
**BMI at diagnosis (kg/m2)**	25 (20;38)
**Body weight before start of semaglutide (kg)**	116 (76;175)
**BMI before start of semaglutide (kg/m2)**	38 (28;58)

Data are given as number (percent) or median (range)

1) One patient with Type 1 Diabetes, six patients with Type 2 Diabetes

**Fig. 1:**
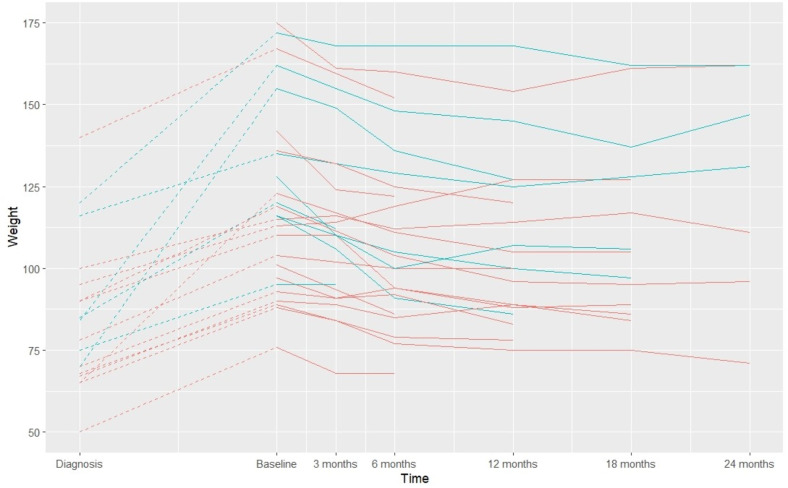
Weight trajectories total cohort. Figure showing body weight trajectories from diagnosis of hypothalamic tumor to initiation of semaglutide treatment and follow-up at approximately 3, 6, 12, 18 and 24 months, respectively. Red lines indicate patients not previously treated with GLP-1 analogues; blue lines indicate patients previously treated with liraglutide. Note that the time point of diagnosis is only illustrative as it differs between patients (see Table 1 for details). In those patients diagnosed in childhood, only baseline and follow-up weight are shown

As illustrated in Fig. 1, patients experienced a massive weight gain (median 25 kg (range 15–85 kg)) from time of diagnosis to initiation of semaglutide treatment. All, but one patient, managed to lose weight during the observation period. The body weight (in kg and in % of baseline weight) as well as BMI decreased significantly compared to baseline at all time points, but seemed to stabilize after 12 months of treatment (Table 2; Fig. 1). However, number of patients were small after 12 months, especially at 24 months, possibly affecting the estimates. The maximum achieved weight loss was 26% of the baseline body weight at 12 months (data not shown). Fifteen patients (58%) lost more than 10% and two patients (8%) lost more than 20% of initial body weight, respectively. There was no difference in weight loss between patients previously treated with liraglutide (*N* = 9) and patients naïve to GLP-1 analogues (*p* = 0.18). We further did not find any significant differences in weight loss response between men (*N* = 11) and women (*p* = 0.86), between patients with (*N* = 7) or without co-existing diabetes (*p* = 0.82) or between groups of patients below (*N* = 7) or above 1 year from diagnosis to the initiation of semaglutide treatment (*p* = 0,14).

**Table 2:** Weight loss from start of semaglutide treatment until 24 months, end of observation period or death. Data given as median (range) weight loss in kg from baseline, in percentage of baseline weight and in BMI, respectively

Time point	*N*	Weight loss (kg) (95% CI)	Weight loss (%) (95% CI)	BMI loss (kg/m^2^) (95% CI)	*p* (all)
**Baseline**	26	-	-	-	*-*
**3 months**	23	6.1 (3.2–9.1)	4.9 (2.5–7.4)	2.0 (1.1-3.0)	< 0.001
**6 months**	24	10.9 (8.0-13.8)	9.0 (6.6–11.4)	3.5 (2.6–4.5)	< 0.001
**12 months**	20	13.4 (10.3–16.5)	10.8 (8.3–13.4)	4.4 (3.4–5.4)	< 0.001
**18 months**	13	13.4 (9.7–17.0)	10.7 (7.7–13.7)	4.4 (3.2–5.5)	< 0.001
**24 months**	7	12.5 (7.9–17.2)	10.4 (6.6–14.2)	4.1 (2.6–5.6)	< 0.001

Two patients paused and re-started the treatment during follow-up, but still managed an over-all weight loss despite a small increase in weight during the pause. One patient consistently gained weight during the study period despite a dosage of 2.4 mg semaglutide with no obvious lack of adherence to the treatment when checking the redemptions of the prescriptions. All other patients had a lower body weight by the end of the study period compared to baseline.

Two patients withdrew from treatment after 6 months due to gastrointestinal side effects; one of them regained all of the lost weight (8 kg) while the other patient regained approximately half (9 kg) of the achieved weight loss (20 kg). One patient died during follow-up, but the death was un-related to the treatment with semaglutide. Eighteen out of the 26 patients had changes in their hormone substitution therapy during follow-up. Two patients started treatment with growth hormone and one patient started treatment with testosterone during the observation period which might have accelerated the observed weight loss. However, we found no clear pattern relating changes in substitution treatment with changes in body weight, although this was not statistically tested due to small numbers. We further did not test the effect of numbers of affected hormone axes on the weight change as most of the patients had pan-hypopituitarism (Table 1).

## Discussion

In this observational study of real-world evidence, we observed a significant and clinically relevant effect of semaglutide on weight loss in a group of patients with HO due to brain tumors affecting the hypothalamic region. Semaglutide also demonstrated efficacy in weight management in patients previously treated with liraglutide. To our knowledge, this is the largest published real-world data-set on the efficacy of semaglutide on HO.

The only RCT to date exploring GLP-1 analogues for HO was a 36-week study conducted in 2021 including 35 participants completing the study. In this trial patients were randomly assigned to once-weekly exenatide or placebo. The study failed to demonstrate a significant change in BMI between the two treatment arms but found a significant reduction in body fat mass and waist/height ratio in the treated group compared to placebo [18]. Another, much smaller and non-controlled intervention with exenatide found similar results [19]. We believe that the discrepancies in weight loss results between our study and the two studies using exenatide might be explained by the choice of drug. Second-generation GLP-1 analogues, such as semaglutide, relative to first-generation analogues like exenatide or liraglutide, have previously shown enhanced efficacy compared to other GLP-1 analogues [24–26]. The underlying mechanisms distinguishing the effects of different GLP-1 analogues remain controversial. Studies have shown that semaglutide (and liraglutide) in contrast to exendin-4 do not cross the blood-brain barrier (BBB) [27, 28]. However, as the hypothalamus and hindbrain lack BBB, semaglutide might act directly on these structures. Semaglutide has an enhanced binding affinity and stability compared to first-generation GLP-1 analogues due to an amino acid replacement (preventing dipeptidyl peptidase 4 degradation) and a C18 fatty diacid addition [23]. These modifications not only increase the plasma half-life, enabling weekly dosing, but may also enhance the pharmacodynamic properties of the drug, possibly accounting for some of the differences in reported effects between GLP-1 analogues.

Six case reports focusing on either a single individual or very few individuals with HO found results similar to our study with substantial weight reduction accompanying treatment with GLP-1 analogues. Most of these studies used liraglutide, but also exendin-4, dulaglutide, exenatide and semaglutide were used [13–17, 20].

Pharmacological treatments for acquired HO, besides GLP-1 receptor agonists, have previously been investigated, of which the most promising include treatment with methionine aminopeptidase inhibitors [29], dextroamphetamine [30, 31] and Tesomet (tesofensine combined with metoprolol) [32]. Of these, the largest study with the longest follow-up was the randomized controlled trial of Tesomet treatment versus placebo in 21 patients with acquired HO over 24 weeks showing a mean weight loss of 6.3% with mild, although significant, side-effects such as headache, sleep-disturbances and dry mouth [32]. Other approaches to treat HO have been limited by the lack of effect or serious side-effects [33–36].

From larger RCTs on semaglutide in patients with Type 2 diabetes as well as in people with obesity without Type 2 diabetes there is substantial evidence demonstrating the robust effects on body weight reduction [9, 37–40]. In studies of common obesity, semaglutide has shown larger effect sizes than we found in our data while the reported weight loss in subjects with Type 2 Diabetes has been slightly lower [9, 37–40]. We do not have an exact explanation of these differences. However, we are not surprised to find effect sizes within the previously reported range as our patient group was quite heterogenous regarding degree of obesity, dosage of semaglutide and presence of diabetes making a direct comparison with RCTs on common obesity or Type 2 Diabetes difficult. It is noteworthy that those patients who withdraw from treatment due to gastro-intestinal side effects or the cost of the medication (only partly covered by subsidy) all rapidly re-gained weight (data not shown), underlining the importance of continuous treatment for sustained weight management as previously reported [41].

The effect of semaglutide on body weight regulation is known to be partly mediated by regulation of the hypothalamic appetite center. However, in patients with HO arising from brain tumors or surgical or irradiation damage, it is unlikely that the GLP-1 receptors in hypothalamus remain completely intact. We speculate that residual response in the arcuate nucleus might still be present, particularly under supra-physiological dosages of GLP-1 RA. One case report from 2020 showing effect of high-dose liraglutide after failed response to gastric bypass in a patient with craniopharyngioma supports this suggestion [14].

Another explanation of the treatment response could be due to extra-hypothalamic effects of the GLP-1 analogues. A previous study found a significant association between the extent of hypothalamic damage as visualized by Magnetic Resonance Imaging and the response to GLP-1 analogue treatment (BMI and body fat). This association is hypothesized to reflect increased extra-hypothalamic effects in the presence of more extensive hypothalamic damage [42]. Some of these effects are thought to be related to areas in the brain stem and vagus nerve. Other extrahypothalamic effects of GLP-1 have been identified in the orbitofrontal cortex, the striatum, the area postrema and the insula [43, 44]. Finally, the well-established local effect of GLP-1 in the intestinal tract may also contribute to weight loss in acquired HO.

Despite the fact that many substitution therapies are considered weight dependent, we did not find any specific pattern in dosage changes of the substitution therapy during the observation time reflecting the many different factors affecting the hormone levels in a real-life setting.

The strengths of this study are the relatively large number of participants, the relatively long follow-up time and the real-life setting demonstrating the feasibility of the clinical implementation of the treatment. However, due to the retrospective real-life data design, several limitations also apply. The primary limitation of our study was the absence of a placebo control group. However, we found a much greater weight loss on semaglutide than reported in the before-mentioned RCT investigating exenatide [7], even without further weight loss support or dietician counselling. Thus, we find it unlikely that the massive weight loss in our patients is solely attributed to a placebo effect or confounding. We did not have systematical data on (changes in) hyperphagia nor the presence of nausea and other side-effects which is an important limitation to the study compared to most RCTs. Only two patients (8%) stopped the treatment due to intolerable side effects which is comparable to previous findings [40]. The median dosage of semaglutide of 1.6 mg in our data was lower than in most weight loss trials, but we do not have evidence of side-effects as the main cause of not achieving the maximum dosage. Furthermore, we did not have measures of the quality of the weight loss regarding changes in waist circumference and/or body composition which are important in terms of metabolic health. Finally, the patient group was rather heterogenous in terms of degree of obesity and duration of the disease, reflecting the real-life setting. We did not have data to assess whether obesity occurred primarily before or after surgery of the patient and/or whether early intervention with semaglutide would have made any difference to the weight loss response. We tried to address this question by adding a co-variate of disease duration before start of semaglutide to the model, but did not find any significant difference. To further address such questions, we believe, there is a need for large (multi-center) RCTs, however, the rarity of HO, make such RCTs difficult and time costly.

In conclusion, treatment with semaglutide in a daily clinical setting demonstrated good feasibility and efficacy in patients with HO who historically have been challenging to treat for significant weight gain. Thus, the study highlights the potential of semaglutide as a viable treatment option for managing the profound weight issues in this patient group. The field of medical treatment for obesity is entering an exciting new era (e.g. development of dual and tri-agonists treatments). This advancement holds significant promise for patients with obesity including patients suffering from HO.

## Data Availability

No datasets were generated or analysed during the current study.

## Declarations

### Reviewer suggestions

Gudmundur Johansson, Professor at the Institute of Medicine, University of Gothenburg, gudmundur.johannsson@medic.gu.se. Pia Burman, Physician at Translational Muscle Research, Lund University, pia.burman@med.lu.se.
